# Qualitative study of the impact of an authentic electronic portfolio in undergraduate medical education

**DOI:** 10.1186/s12909-014-0265-2

**Published:** 2014-12-17

**Authors:** Rosie Belcher, Anna Jones, Laura-Jane Smith, Tim Vincent, Sindhu Bhaarrati Naidu, Julia Montgomery, Inam Haq, Deborah Gill

**Affiliations:** Department of Medical Education, University College London Medical School, London, UK; Brighton and Sussex Medical School, University of Sussex, Brighton, UK

**Keywords:** Portfolio, Assessment, Feedback, Transition, Enculturation

## Abstract

**Background:**

Portfolios are increasingly used in undergraduate and postgraduate medical education. Four medical schools have collaborated with an established NHS electronic portfolio provider to develop and implement an authentic professional electronic portfolio for undergraduate students. We hypothesized that using an authentic portfolio would have significant advantages for students, particularly in familiarizing them with the tool many will continue to use for years after graduation. This paper describes the early evaluation of this undergraduate portfolio at two participating medical schools.

**Methods:**

To gather data, a questionnaire survey with extensive free text comments was used at School 1, and three focus groups were held at School 2. This paper reports thematic analysis of students’ opinions expressed in the free text comments and focus groups.

**Results:**

Five main themes, common across both schools were identified. These concerned the purpose, use and acceptability of the portfolio, advantages of and barriers to the use of the portfolio, and the impacts on both learning and professional identity.

**Conclusions:**

An authentic portfolio mitigated some of the negative aspects of using a portfolio, and had a positive effect on students’ perception of themselves as becoming past of the profession. However, significant barriers to portfolio use remained, including a lack of understanding of the purpose of a portfolio and a perceived damaging effect on feedback.

**Electronic supplementary material:**

The online version of this article (doi:10.1186/s12909-014-0265-2) contains supplementary material, which is available to authorized users.

## Background

Recent moves towards competency-based medical education [1,2] and an increased emphasis on reflective practice [3] have led to an expansion in the use of portfolios in education for the health professions. There has been widespread adoption for training, continuing professional development and most recently, revalidation across the postgraduate sector. In the UK, the General Medical Council (GMC) now requires medical students to ‘establish the principles of life-long learning including a professional development portfolio containing reflections, achievements and learning needs’ [2].

Portfolios have been claimed to support competence-based medical education as they support the longitudinal recording of evidence of experience and achievements, feedback received, and future plans [4,5]. Most authors also agree that demonstrating reflection on experience is an important function of a portfolio and that portfolios are well placed to promote this as they can combine reflection on experience with self assessment and external assessment [4-6].

However, portfolios have met with mixed success in practice [4]. One systematic review identified increased self awareness and engagement in reflection and improved feedback to students, but several studies within this review questioned the quality of the reflection undertaken, and also noted that the time commitment was perceived as burdensome and distracting from clinical learning [7].

### The Undergraduate Medical ePortfolio (UMeP) Project

Since 2010, four UK medical schools have collaborated with a major National Health Service (NHS) portfolio provider to create an electronic portfolio for medical students, the Undergraduate Medical ePortfolio (UMeP). The UMeP is adapted from the ePortfolio used by the majority of Foundation Programme doctors in the UK. The Foundation Programme refers to the first two years of training after graduation. In order to meet the specific needs of the curriculum, a number of institutions have developed bespoke tools, but these are often expensive to develop and maintain, and it may be difficult to access or transfer data to other applications at a later date. Although adapting an existing postgraduate portfolio for use by undergraduates introduces some limitations, particularly in reducing flexibility to adapt to individual institutional needs, we felt that there are two significant advantages to using an authentic portfolio that would outweigh these potential limitations.

Firstly, students have reported that using a portfolio as an undergraduate increased their confidence to use a portfolio in the future [6], and we hypothesised that being accustomed to the ePortfolio and its assessments from early on would ease the stressful transition into the Foundation Programme. Secondly, we hypothesised that using the UMeP would prepare students for a career of assessment through portfolios, where assessment and reflection are integral. Lastly, we also hypothesised that using an authentic portfolio early in their careers would mitigate some of the negative perceptions of portfolios, particularly that they distract students from clinical learning, as students would be able to compare their use to use by doctors.

This paper describes part of the evaluation of the UMeP in two of these schools, with a focus on our last hypothesis. This paper cannot comment on the first hypothesis as our first cohort of students to use the UMeP has just entered the Foundation Programme (Aug 2014).

The UMeP is adapted from the ePortfolio used by most Foundation doctors, and has the same appearance and structure as this ePortfolio. Some features (eg reflective and personal development plan (PDP) logs) are identical to the versions used by Foundation doctors and are available to all student users. The schools have collaborated to create student-specific versions of some elements (eg a multisource feedback tool), which are then available for use without further adaptation by schools. Other elements which contain school-specific information (eg forms used for recording structured learning events or module feedback) are individually adaptable for each school from generic templates. The schools meet regularly to discuss the development and management of the UMeP, and have agreed to keep the structure, appearance and features as similar as possible to the Foundation ePortfolio.

At both schools the UMeP was introduced to students entering their first year of predominantly clinical placements, but the schools differ in their use of the UMeP. At school 1, the UMeP is used mainly to encourage reflective practice and to support the student’s relationship with their Clinical Academic Tutor (CAT) through recording meetings. Other elements of the UMeP such as the ability to upload additional documents (the “personal library”), and PDP logs are also available to students. Using the UMeP at this school is voluntary.

Students at school 2 are required to use the UMeP to record work-based activities such as structured learning events (SLEs), module report forms, and meetings with their Personal Tutor. Students are also encouraged to use the non-compulsory elements including reflective and PDP logs, and personal library.

Information entered in the UMeP can be seen by both the student, their CAT (school 1) or Personal Tutor (school 2), and the head of year, but not to those entering other assessments via an electronic ticketing system. In addition, reflective logs can be entered as “private”, when they are available only to the student.

At both schools, the purpose and broad aims of the UMeP was introduced to students in lectures at the start of the year, and detailed, institution-specific guidance was available online. In addition, both schools appointed student “advocates” who attended an additional training evening on the purpose and use of the UMeP. Students were encouraged to contact advocates in the first instance if they had problems, and advocates had direct access to staff for more complex queries. Teachers had mostly encountered the ePortfolio through their supervision of Foundation doctors, and were given general information about the change to the UMeP.

### Aims

This study sought to explore students’ attitudes towards and experience of using the UMeP. The research questions focused on students’ understandings of the purpose and impact of the UMeP in their education and factors that act as facilitators or barriers to using it. The study was conducted across two schools to explore contextual impacts on students’ views, and to gather opinion on experiences of students using different functions of the UMeP.

## Methods

We adopted a constructionist epistemology during this research, which asserts that people construct knowledge through their interactions with one another and the world. We chose this framework as we are particularly interested in how students formed their opinions through their interactions with supervisors and junior doctors, and on how the different contexts of portfolio use influenced students’ opinions. Within this framework, we undertook thematic analysis of the data. Thematic analysis is a method for identifying and organizing patterns in qualitative data [8]. The analysis was inductive, allowing the data rather than theory to drive coding.

All student users in both schools were invited to take part in this study, which was conducted at the end of the first year of using the UMeP. Researchers in both schools agreed the focus of questions to be used with students to address the research question. In school 1 use of the UMeP is voluntary, and for this reason a questionnaire was used to gather opinions from both users and non-users (See additional file 1 for questionnaire). The questionnaire was developed by the authors and piloted with a group of student volunteers. The response rate was high (111/125, 90%). It included significant free text sections inviting extended comments, and it was these free text comments that were used as data in this study.

At school 2 the UMeP is compulsory and purposive sampling was used to form focus groups (See additional file 2 for focus group topic guide). Three focus groups were held, with a total of 15 participants. Participants included advocates, and students with both high and low levels of engagement with the UMeP. The discussion followed semi-structured guides, which were developed by the authors mindful of the questions used by school 1 and based on the research question outlined above. Saturation of themes was reached after the third focus group. Focus groups were audio recorded and professionally transcribed.

Participation by students in the evaluation was voluntary at both schools. The Research Ethics Committee (REC) at School 1 granted approval for the study. At School 2, the project was exempt from formal ethical review following discussion with the University REC chair.

### Data analysis

Thematic analysis was used to analyse the collated comments and focus group data from both schools. Individual researchers examined the transcripts and collated comments iteratively, and created an initial coding structure. This initial coding was conducted separately within each school. Researchers within each school then met to review the data and emerging themes, compare and agree upon common themes, and identify themes particular to each setting. These initial themes were then taken back to a small number of student users of the UMeP on each site for participant validation, and a final coding structure and themes were agreed.

## Results

Five major themes were identified from the focus groups and free text comments data and are described below:

1. Purpose, use and acceptability
2. Advantages of using the UMeP
3. Barriers to the use of the UMeP
4. Impact on learning
5. Professional identity

Numbers in brackets following quotes indicate which school the participant is from.

### Purpose and use of the ePortfolio

As might be expected, students’ views varied on the role and purpose of the UMeP, and this was also coloured by how the School was using the UMeP and the School’s stance with regards to the requirement to engage. At School 1, where engagement is voluntary and use limited to reflective practice and supporting tutor meetings, just over half of participants believed they understood the purpose of a portfolio in lifelong learning. A third of participants at School 1 had not used the UMeP at the time of the study and the majority of these non-users claimed not to understand the purpose. Indeed when non-users were asked why they had not yet used the UMeP, the most common response was that they were not sure what to use it for.

As engagement with the ePortfolio was compulsory at School 2, all students had used the UMeP and students were clearer about the purpose. They suggested a range of purposes including providing feedback, continuous assessment, and as a record of their development.

*“It’s a way of… keeping track… of what we’ve done. Assessment throughout the year.” (2)*

*“To show your professional development really.” (2)*

*“It’s like a log book for sort of learning and progress right from the start in clinical teaching.” (2)*

*“It’s supposed to be so that doctors can give students or other doctors feedback about clinical skills and how they’ve been doing.” (2)*

Furthermore, in School 2 the UMeP is used to record SLEs, and participants acknowledged the UMeP was driving them to practice and seek feedback on their clinical skills and performance, something they felt they might have otherwise neglected.

*“It does keep you on your toes, it gives you a kind of target to work towards… I think some students might… be a bit lost in terms of what do I need to get out of this. And if they’ve had two supervised examinations and two history presentations it’s good, because in the process they’ve… had the practice.”* (2)

The idea of using the UMeP to facilitate reflection gathered mixed responses from students, with some appreciating the opportunity, but many resisting the UMeP as a reflective tool.

*“Reflection and writing about experiences is a good way to learn, but doing so on the ePortfolio seems artificial.” (1)*

*“When I have reflected I’ve kind of put it in my own personal notes… I’ve found the website a bit difficult” (2)*

### Advantages of using the UMeP

Students identified a number of advantages to the use of portfolios in general and specifically electronic portfolios. They noted that assessment drives learning and in School 2 where work-based assessments were integral, the tool particularly encouraged workplace-based learning.

*“Especially if you’re coming towards exam time and you know that you have to get stuff [SLEs] done, you’ll have to go into clinics, or hospital and get them done … [otherwise] you’re most likely going to be revising. And okay that’s going to help you with the exams, but then it’s not really going to help you as an F1” (2)*

The majority of participants at both schools stated that they preferred a web-based portfolio to a paper portfolio. The major advantages of an electronic portfolio were identified as durability and accessibility.

*"I know that if I ever lost it or you know everything got burnt in a fire… I would have everything there all listed in my ePortfolio." (2)*

*"It's convenient that you can access it from anywhere" (2)*

### Barriers

A number of potential barriers to accessing and using the UMeP were identified. These included a lack of engagement, software issues, the use of their data, accessibility and the ’tick box’ culture.

#### Engagement of faculty and healthcare colleagues

Students at both schools suggested that a lack of faculty engagement with the UMeP was a significant barrier to its use:

*“When the consultants didn’t really see what the point of it was, it was very difficult to use, there was a lot of friction. Whereas when the consultants were fine with it and kind of saw it as part of the way you did things, then it was great” (2)*

As suggested in this comment, the attitude of faculty was highly variable, and some students reported that some supervisors did not engage due to the electronic nature of the portfolio.

*“I’ve had some doctors say to me ‘if it’s on the computer I’m just not going anywhere near it’” (2)*

Students’ comments suggested that although some supervisors were initially surprised to find students using the UMeP, in the main problems with engagement were overwhelmingly related to attitude rather than lack of knowledge of what was required, as

*“Everyone kind of knows the system, knows how it works.” (2)*

#### Potential use of data

Students at both schools also raised concerns about security of information, and in particular the tension between wanting to use the reflective practice logs to undertake meaningful reflection as a formative learning process, while being concerned about the negative impact of the use of such reflections later, if the ePortfolio were to be used for summative or showcase purposes.

*“Fear of accidently doing something irreversible and wrong which stays” (1)*

*“Then I was like ‘Well how private is this?’ You know if the GMC wanted to see this?… so I basically deleted everything” (2)*

Students also expressed concern about whether material they had entered as a student would be available to supervisors in the Foundation Programme, or to interviewers.

#### Accessibility and feedback

Students at School 2 also identified a cluster of issues around the accessibility of the UMeP in the workplace, which detracted from its ability to record SLEs. Particularly in hospital settings, it was difficult for students to find a computer to complete the assessment immediately, requiring them to send an electronic ticket to the assessor to be completed later. They felt this disconnect between performance of the assessment and completion of written feedback contributed to poor feedback, as some supervisors waited a considerable time before completing the online feedback, when they then struggled to remember individual students.

*“Some of the clinicians started talking to me, like ‘We get a few of these every day, and there’s no way that you can just write your name and we’re going to remember you’ ” (2)*

The majority of students in School 1 were accessing the UMeP at home so these issues did not arise.

#### The tick box culture

Students at both schools identified a further disincentive to use related to practice. Whilst they understood the purpose of recording their learning, they found the way the UMeP was used in practice sometimes undermined the intended purposes. In particular, students felt that it easily became a “tick box” (2) or “hoop jumping” (2) exercise.

*“We will just do it to get it done and meet deadlines, rather than for its actual purpose” (1)*

#### Impact on learning

Students found it hard to identify or articulate meaningful impacts of the UMeP on their learning. Students at School 2 using the UMeP for SLEs felt that the quality of feedback they received meant they did not see an impact on learning. The lack of usefulness of the feedback was due to the delay between any observation and subsequent completion of the UMeP form, the rigidity of the forms used and a lack of engagement of their supervisors with the feedback process.

*“I think the feedback is often a bit weaker when they do it at a later date. If you get the feedback there and then, they’re more likely to remember what you did and pick up on stuff.” (2)*

*“[Doctors] can’t be bothered typing more than a few words really in their evaluation” (2)*

Since the feedback received was perceived to be either poor-quality or non-existent, students confessed that they did not always read their feedback, and that this was a further contributing factor to the perception of the UMeP as a “tick box exercise”.

In School 1 where the UMeP is not used for formative assessment purposes, participants also struggled to identify learning outcomes. However, several of the responses hinted that they could see a potentially beneficial effect on learning in the future from current engagement and some participants identified that *not* being used for assessment was beneficial in terms of their learning:

*“Less pressure because not assessed so feel my reflections have been more meaningful as I can wait until I have actually had an interesting, thought provoking experience.” (1)*

### Professional identity and authenticity

Students at both schools indicated that they thought it likely that using the ePortfolio as students would assist them as Foundation doctors, although these opinions were often qualified by comments about the time needed to maintain a portfolio.

*“The advantage it gives us is that obviously you have it when you’re a junior doctor and probably forever, so at least we get used to it now… and we know how to use it. Which means that when we actually qualify it’s not this other new thing we have to get our heads around.” (2)*

*“The key reasons I haven't used the ePortfolio much despite knowing how useful it will be for the future is simply because of a lack of time.” (1)*

Students at School 2 were aware of, and commented extensively on the use of portfolios by doctors, and felt that participating in portfolio keeping contributed to their own enculturation into the profession. Their comments suggested their shared frustrations with the ePortfolio helped forge relationships with senior colleagues.

*“I think at first a lot of students just thought this is something that the med school is doing and it’s not really related to our careers in the future. Whereas once you actually start speaking to junior doctors and you hear that this is what they’re doing … then you start thinking ‘Okay there is a point to this’” (2)*

*“I think it kind of helps you bond with them [junior doctors].” (2)*

By contrast, at School 1, less than half of participants had discussed the ePortfolio with Foundation doctors, and their comments suggested they are less aware of how doctors use portfolios:

*“[We would like an] explanation of what we would use it for as a Foundation doctor, so we can see the relevance.” (1)*

*“I’ve never seen any doctors (or any rank) use ePortfolio. So I feel it isn’t high on the list of things to do” (1)*

## Discussion

The recent introduction of revalidation in the UK, which is based on the evaluation of a portfolio of evidence, means that, as one of our students put it, portfolios are here “probably forever”. Students at both schools in our study acknowledged this and thought that familiarity with the tool the majority of them will use as Foundation doctors would be helpful to them. Our first cohort of students to use the UMeP throughout their clinical undergraduate years have graduated in 2014, and we plan further research on the impact of early use on their experience of the ePortfolio as Foundation doctors.

Other studies have suggested that undergraduate students perceived maintaining portfolios as detracting from clinical learning [9,10]. Our students did not report this, and in fact several reported that completing assessments and maintaining the ePortfolio encouraged them to gain clinical experiences they might otherwise have missed. This supports our hypothesis that using an authentic portfolio can mitigate some of the negative perceptions of maintaining a portfolio. However, some negative aspects remained, since some of the challenges associated with the UMeP caused students to feel that using it was a time consuming process and that feedback on SLEs did not help their learning. Postgraduate trainees have also expressed this opinion [11-13], although when directly questioned, students in our study believed that they developed this attitude independently from the influence of junior doctors.

The authenticity of the portfolio also had positive effects on students’ perception of themselves as becoming part of the profession, which we had not predicted. This occurred mainly at school 1 where use of the UMeP more closely mirrored use of the ePortfolio by Foundation doctors, and the requirement for students to ask their supervisors to complete assessments probably resulted in more discussions of the ePortfolio in general. We think this encouraged students to draw parallels between their current and future use, and helped them to see their future selves as users of the ePortfolio. We would suggest that educators thinking of introducing a similar initiative for undergraduates should consider following as closely as possible the portfolios use for postgraduates in order to take most advantage of this effect.

In common with junior doctors [13,14], students in our study sometimes struggled to understand the purpose of maintaining a portfolio. Again, comparison between the schools suggests that more closely following the use in postgraduate practice, in particular making portfolio use a requirement rather than an option improves students’ understanding of the purpose. Other studies have reported an association between positive perceptions of a portfolio and better engagement [11], but it is intriguing that our comparison suggests that understanding of the purpose comes from engagement, not vice versa.

Some of the challenges and barriers to use were particular to this ePortfolio. The practical accessibility of the UMeP in the workplace is crucial to meaningful feedback, and students felt the delays contributed significantly to poor quality feedback. The ePortfolio does not yet have an app to support contemporaneous assessment on a mobile device, and although some students had entered assessments on a mobile phone, this was difficult and time consuming. In response to this, School 2 has recently provided students with tablet computers, and will evaluate the impact on students’ opinions of the utility of SLEs and the feedback that they receive.

Some of the barriers to use are related to more general issues with portfolios, and have been reported by others in relation to portfolios. As with postgraduate portfolios, supervisor engagement is a significant determinant of the ease of use of the portfolio for students, and the usefulness of feedback received [15]. Students reported a range of attitudes from supervisors, and the attitude of supervisors could transform an experience of “friction” in to “the way you d[o] things”. It is concerning therefore that studies of supervisors’ opinions on portfolios have suggested that supervisors often hold negative opinions of the educational value of a portfolio [16], (or perhaps worse, marked indifference [17]) and that trainees report that a significant proportion of educational supervisors do not fulfil all their duties [18]. Further investigation of supervisors’ opinions of how the educational impact of the ePortfolio could be improved, and better selection, education and training of supervisors could help improve students’ engagement with the ePortfolio.

The negative effects on feedback were unexpected although others have also found that electronic portfolios can alter the dynamics for teachers when giving students feedback [19]. Other examples in healthcare (eg [20]) emphasize that the change from paper to electronic formats is not a simple transition but in fact transforms processes and structures with profound consequences [21].

A significant source of tension for students concerns the use of the information in the ePortfolio. Students who had used the UMeP for reflection were anxious about how honesty, particularly if it revealed a personal weakness or mistake, could count against them later if the ePortfolio were used for more summative purposes. Foundation doctors have raised similar concerns [22]. Our first cohort of student ePortfolio users has just entered the Foundation programme, and currently, information recorded against their student account remains visible to the student but not available to Foundation supervisors. This remains under review.

A systematic review of the educational effects of portfolios reported that most studies reported positive effects on student learning with few reporting neutral or negative effects [7]. This seems at odds with the often negative or cynical attitudes encountered to portfolios [11,12], and we have highlighted in our evaluation that although there were positive aspects, in particular the effect on student socialisation into professional roles, there were also significant negatives, including the perceived damaging effect on feedback.

### Strengths and limitations of this study

The strengths of this study are that the evaluation across two different medical schools has allowed us to compare responses from students using the UMeP in different contexts and for different purposes, and the inclusion of student users in interpreting the data broadens the perspectives available.

One limitation of this study is that the use of different methods in the evaluation potentially limits the extent to which we can draw conclusions and develop recommendations from this data. Data obtained from written questionnaire answers may be less contextually rich than that obtained in focus groups; however, it is recognised that focus group participants may not be representative of their colleagues and therefore data from a survey with a high response rate is a useful addition.

## Conclusion

This evaluation has explored the impact on students of using an authentic portfolio. It has demonstrated that, whilst there are barriers to its use, students also recognise the potential advantages, and the positive impact on professional enculturation. Students constructed their opinions of the ePortfolio from multiple sources, and were influenced significantly by the supervisors and junior doctors they encountered, as well as their own experiences.

Providing our students with early exposure to the portfolio tool that they are likely to use as Foundation doctors will, we anticipate, support them in their transition from medical student to doctor. Our first cohort of students, who have used the ePortfolio throughout their clinical training have now graduated, and we plan further collaborative research to follow them in to the Foundation programme, and investigate the effects of using an authentic portfolio as a student.

## Additional files

Additional file 1:
**Questionnaire used at School 1.**
Additional file 2:
**Focus group topic guide used at School 2.**

## Abbreviations

CAT: Clinical Academic Tutor
GMC: General Medical Council
PDP: Personal Development Plan
REC: Research Ethics Committee
SLE: Structured Learning Event
UMeP: Undergraduate Medical ePortfolio
