# Supplementary material for: Qualitative study of the impact of an authentic electronic portfolio in undergraduate medical education
Source: BMC Med Educ. 2014 Dec 17;14:265. doi: 10.1186/s12909-014-0265-2 (PMC4272766; doi:10.1186/s12909-014-0265-2)
Supplement: Additional file 1: — Questionnaire used at School 1. [file 12909_2014_265_MOESM1_ESM.docx]

# Additional file 1. Questionnaire (School 1)

Q1. Have you used any functions of the ePortfolio?

Yes □ – proceed to Q2 No □ – please go directly to Q4

Q2. Using the ePortfolio:

Please indicate how you would describe your experience of using the ePortfolio:

| Very easy | Easy | Neutral | Difficult | Very difficult |
| --- | --- | --- | --- | --- |
| 1 | 2 | 3 | 4 | 5 |

Q3. Use of different functions of ePortfolio:

Please indicate which of the main sections of the ePortfolio you have used so far:

|  | Used |
| --- | --- |
| Clinical academic tutor (CAT) meeting form |  |
| Personal development plan (PDP) |  |
| Personal library |  |
| Reflective logs |  |
| Self-appraisal |  |
| Uploaded forms eg CbD feedback |  |

Q4. Reasons for *not* using the ePortfolio:

If you answered *No* to Q1 and have not as yet used the ePortfolio in any way, we would like to understand more about why this is:

*Tick any that are applicable – you may tick more than one.*

| Reasons for non-use of the ePortfolio: | |
| --- | --- |
| I didn’t know about it |  |
| I would like to use it but have not been able to log in |  |
| I am not sure what I should be using it for |  |
| I don’t see how it would be useful for me |  |
| I am concerned about confidentiality and the security of my data |  |
| It’s not compulsory or assessed so I haven’t used it |  |
| My CAT hasn’t mentioned it or advised me to use it |  |
| I know I’ll have to use it as a Foundation doctor but don’t see how it’s relevant to me now as a student |  |
| I haven’t had time to use it |  |
| My friends aren’t using it so I haven’t either |  |
| I don’t want to use it because.....  .......................................................................................................... |  |

Q5. What would persuade you to use the ePortfolio in the future?

*Tick any that are applicable – you may tick more than one*

| Making it assessed / compulsory |  |
| --- | --- |
| Providing me with more support and guidance on how to use it |  |
| Providing me with more evidence on how this will support my training |  |
| Hearing from junior doctors as to how they have used this in their training |  |

Please complete *all* of the following questions *whether or not* you have used the ePortfolio:

Q6. How satisfied were you with the guidance that was been provided to you about the portfolio at the start of the year?

| Satisfied |  | Neutral |  | Dissatisfied |
| --- | --- | --- | --- | --- |
| 1 |  | 2 |  | 3 |

Q7. Have you looked at the guidance in the ePortfolio section of studentcentral to assist you in using the ePortfolio.

Yes □ – go to Q8 No □ – please go to Q 9

Q8. Please indicate your agreement with the following statement:

“The information contained in the ePortfolio section on studentcentral has been useful in helping me to use the ePortfolio”

| Strongly agree | Agree | Don’t know | Disagree | Strongly disagree |
| --- | --- | --- | --- | --- |
| 1 | 2 | 3 | 4 | 5 |

Q9. “I understand the purpose of using a portfolio and its role in life-long learning”

| Strongly agree | Agree | Don’t know | Disagree | Strongly disagree |
| --- | --- | --- | --- | --- |
| 1 | 2 | 3 | 4 | 5 |

Q10. “Reflecting on and writing about interesting clinical cases helps me to identify my educational needs”

| Strongly agree | Agree | Don’t know | Disagree | Strongly disagree |
| --- | --- | --- | --- | --- |
| 1 | 2 | 3 | 4 | 5 |

Q11. “I prefer using a web based ePortfolio rather than a paper portfolio”

| Strongly agree | Agree | Don’t know | Disagree | Strongly disagree |
| --- | --- | --- | --- | --- |
| 1 | 2 | 3 | 4 | 5 |

Q12. “The ePortfolio should be compulsory for all year 3 and above students”

| Agree |  | Don’t know |  | Disagree |
| --- | --- | --- | --- | --- |
| 1 | 2 | 3 | 4 | 5 |

Q13. “In the future the portfolio should be used for assessment purposes such as mini-CEX”

| Agree |  | Don’t know |  | Disagree |
| --- | --- | --- | --- | --- |
| 1 | 2 | 3 | 4 | 5 |

Q14. “I anticipate that using the ePortfolio as an undergraduate student would make it easier for me to use it as a foundation doctor”

| Agree |  | Don’t know |  | Disagree |
| --- | --- | --- | --- | --- |
| 1 | 2 | 3 | 4 | 5 |

Q15. What further information or support would help your use of the portfolio?

Is there anything that you think that we should do differently for the next Year 3 group when introducing it to them?

Q16. Any further comments on the ePortfolio?
