# Supplementary material for: Qualitative study of the impact of an authentic electronic portfolio in undergraduate medical education
Source: BMC Med Educ. 2014 Dec 17;14:265. doi: 10.1186/s12909-014-0265-2 (PMC4272766; doi:10.1186/s12909-014-0265-2)
Supplement: Additional file 2: — Focus group topic guide used at School 2. [file 12909_2014_265_MOESM2_ESM.docx]

### Additional file 2. Focus group topic guide (School 2)

### Views and attitudes on the ePortfolio

1. What is your understanding of the purpose of an ePortfolio?
2. What have you found to be the advantages of using the ePortfolio? How do you think your experience of using the ePortfolio at this stage in your training could change your use of it in the future?
3. What have you found to be the disadvantages of using the ePortfolio?
4. Do you have any concerns or anxieties about using the ePortfolio?

### Attitudes to and experience of the implementation of the ePortfolio

1. Did staff support you to use the ePortfolio? If so, how?
2. Did you encounter any difficulties in using the ePortfolio? If so, what difficulties did you encounter and how did you overcome these?
3. What should we consider doing differently next time? And what information or support would you liked to have had?

### Use of the ePortfolio

1. How have you used the ePortfolio? Have you used any of the non-compulsory elements such as the reflective logs? Why/why not?
2. What did you like most about using the ePortfolio?
3. Which section of the ePortfolio do you find most useful?
4. Is there anything else that you would like to see included in the ePortfolio?
5. How has the attitudes of your peers affected your feelings about using the ePortfolio?
6. How has the attitude of your teachers affected your feelings about the ePortfolio?
7. How has the attitude of the junior doctors affected your feelings about the ePortfolio?

### Technical issues

1. What do you think about the look and feel of ePortfolio? Compared to other software you use in your personal and professional life, how does the ePortfolio compare?
2. What ideas do you have for improving the technical aspects of ePortfolio?
